# Effect of Ovariectomy on Stimulating Intracortical Remodeling in Rats

**DOI:** 10.1155/2014/421431

**Published:** 2014-09-21

**Authors:** Chun Lei Li, Xi Ling Liu, Wei Xin Cai, Weijia William Lu, Roger A. Zwahlen, Li Wu Zheng

**Affiliations:** ^1^Discipline of Oral Diagnosis & Polyclinics, Faculty of Dentistry, The University of Hong Kong, Prince Philip Dental Hospital, 34 Hospital Road, Hong Kong; ^2^Discipline of Oral and Maxillofacial Surgery, Faculty of Dentistry, The University of Hong Kong, Hong Kong; ^3^Department of Orthopedics & Traumatology, Li Ka Shing Faculty of Medicine, The University of Hong Kong, Hong Kong

## Abstract

*Objective.* Technically primates and dogs represent ideal models to investigate diseases characterized by abnormal intracortical remodeling. High expenses and ethical issues, however, restrict the use of those animals in research. Rodent models have been used as alternatives instead, but their value is limited, if none, because these animals lack intracortical bone remodeling. This study aimed at investigating the effect of ovariectomy onto the stimulation of intracortical remodeling in rat mandibles. *Materials and Methods.* Sixteen 12-week-old Spraque-Dawly (SD) female rats were randomly assigned into two groups, receiving either ovariectomy or sham operation. All the rats were sacrificed 18 weeks postoperatively. The entire mandibles were harvested for microcomputed tomography (micro-CT) and histomorphometric assessments. *Results.* Micro-CT examination showed significantly decreased bone mineral density (0.95 ± 0.01 versus 1.01 ± 0.02 g/cm^3^, *P* < 0.001) and bone volume (65.78 ± 5.45 versus 87.41 ± 4.12%, *P* < 0.001) in ovariectomy group. Histomorphometric assessment detected a sixfold increased intracortical bone remodeling as well as an increased bone modeling in mandibles of ovariectomized rats. *Conclusion.* For the first time, to the authors' knowledge, it was detected that ovariectomy stimulates intracortical remodeling in rat mandibles. This animal model might be of use to study various bone diseases associated with an abnormal intracortical remodeling process.

## 1. Introduction

Bone remodeling plays a key role to maintain the skeletal functional integrity. It is a site and time-specific event combining bone resorption and formation. Higher mammals with the harversian system undergo both cancellous and intracortical bone remodeling, while lower mammals without harversian system, such as mice and rats, do not normally undergo intracortical remodeling [1–3].

Ovariectomized (OVX) rodents are a well-established animal model in osteoporosis studies [4, 5]. Apart from trabecular bone turnover changes [6, 7], recent studies reported that ovariectomy may induce intracortical remodeling in long bones [8, 9]. Whereas Kubek et al. found out that ovariectomy induced intracortical remodeling of jawbones in mice [10], stimulation of intracortical remodeling in rats has not yet been investigated, so far.

The intracortical remodeling rate of human jawbones is around 20 times higher than that of iliac crest [11]. Some bone diseases associated with abnormal bone remodeling such as bisphosphonate related osteonecrosis of the jaws (BRONJ) always preferentially affect jawbones [12]. To study this disease* in vivo*, an adequate animal model therefore is of paramount interest. Even though primates and dogs represent ideal models, high expenses and ethical issues do restrict their use. Rodent models have been widely used to investigate bone diseases, but lack of intracortical bone remodeling [13] limited their value in investigating bone diseases with abnormal bone remodeling.

This study investigated the stimulating effect of ovariectomy onto intracortical bone remodeling in rat mandibles.

## 2. Materials and Methods

### 2.1. Animal Care

The animal experiment was approved by the Committee on Use Live Animal for Teaching and Research, The University of Hong Kong. All rats were held in a 12:12 h light-dark circle with each rat separated in a metal cage at 25 degree Celsius room temperature in the Laboratory Animal Unit of the University of Hong Kong. All animals were allowed free access to water and standard rodent diet. Their general well-being was monitored by a 24 h closed circuit television in the holding room during the entire experimental period.

### 2.2. Ovariectomy (OVX) Surgery

Sixteen 12-week-old, female Spraque-Dawly (SD) rats were randomly assigned into an OVX or a control group, with 8 in each. After a 7-day acclimatization period, the rats of the OVX group underwent a bilateral ovariectomy according to our standardized protocol already published elsewhere [14]. In brief, under general anesthesia, a 2 cm abdominal skin incision was performed. Using artery forceps, the ovarian fat pad was gently grasped and the ovaries were exposed. After removing the bilateral ovaries, the fat pad was repositioned into the abdomen. Wound closure was performed in two layers, adapting the muscle layer with single stitch sutures (Vicryl 5/0, Johnsons and Johnson, Hong Kong) and the skin layer with stainless steel wound clips. The control group animals underwent a similar procedure without removing the ovaries. All rats were given meloxicam (5 mg in 250 mL drinking water, Metacam, Boehringer Ingelheim, Germany) for 5 days postoperatively for pain relief [14].

### 2.3. Fluorochrome Sequential Labeling

Fluorochrome labeling using calcein green (15 mg/kg; C0875, Sigma-Aldrich, Saint Louis Mo, USA) and alizarin complexone (30 mg/kg; A3882, Sigma-Aldrich, Saint Louis, MO, USA) was performed 10 days and 1 day before the sacrifice, respectively. The reagents were dissolved in sodium bicarbonate solution and administrated subcutaneously [15].

### 2.4. Sample Preparation

All rats were scarified 18 weeks postoperatively. Mandibles were harvested entirely and the attached soft tissue was carefully removed. All samples were fixed in 10% neutral buffered formalin solution for 2 days and then transferred to 70% ethanol for further use.

### 2.5. Micro-CT Examination

The changes of trabecular microarchitecture were assessed by micro-CT (SkyScan-1076 X-ray microtomography, SkyScan, Kontich, Belgium) according to the manufacturer's instructions. The X-ray source was supplied with 88 kV voltage and 100 *μ*A electrical current. Three-dimensional (3D) images were obtained at a resolution of 18 *μ*m/pixel. The raw images were reconstructed and analyzed using CTAn software (CTAn, version 1.12.0, SkyScan, Kontich, Belgium).

**Figure 1:**
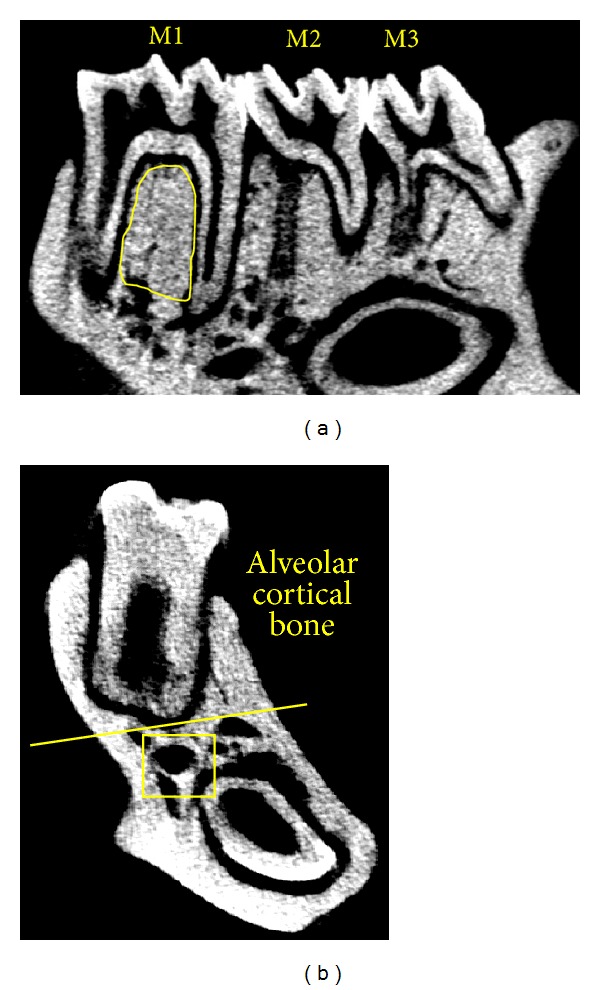
ROI selection in micro-CT and histomorphometry assessment. (a) Trabecular bone inside the circle is selected as the ROI in micro-CT assessment. (b) The bone area above the yellow line is defined as alveolar cortical bone; nonalveolar cortical bone is defined as the area below the yellow line except for trabecular bone (square).

Trabecular bone in the interradicular septum of the first molar (M1) was selected as the region of interest (ROI) (Figure 1(a)). Its microstructure was assessed according to the following parameters: bone mineral density (BMD), bone volume/tissue volume (BV/TV), trabecular thickness (Tb.Th), trabecular number (Tb.N), and trabecular separation (Tb.Sp) [16].

### 2.6. Histomorphometric Assessment

Histomorphometric assessment was performed to evaluate the intracortical remodeling. Nondecalcified mandibles were dehydrated in graded ethanol (70%, 95%, and 100%) and embedded in methyl methacrylate (MMA, Technovit 7500, Kulzer, Hamburg, Germany). Mandibles were sectioned (~100 *μ*m thick) throughout the molar region in a mesial-distal direction using a diamond wire saw. The samples were mounted on slides and ground to a thickness of 80 *μ*m.

To ensure the measurement was performed at a similar region in all the animals, sections containing the deepest root of first molar in each animal were selected. The sections were examined under fluorescence microscope (Nikon, Tokyo, Japan).

#### 2.6.1. Intracortical Remodeling

The alveolar cortical bone was defined as the area above the root at M1 region; nonalveolar cortical bone was defined as the area below the root except for trabecular bone (Figure 1(b)) [10].

To assess the intracortical remodeling, the total bone area (B.Ar), the bone surface (BS), the total length of the labeled bone surface (LS), the single labeled bone surface (sLS), the double labeled bone surface (dLS), and the mean interlabel width (Ir.L.Wi) were measured. The mineralizing bone surface (MS) was calculated as 0.5sLS + dLs. Mineral apposition rate (MAR, *μ*m/day) was calculated as Ir.L.Wi/10 (10 was the interlabel time). For the osteons with only single label, MAR was set as 0.3 [17]. Surface-based bone formation rate (BFR) (*μ*m^3^/*μ*m^2^/day) was calculated as MAR × MS/BS. Intracortical BFR (%/year) was calculated as (MAR × 0.5BS/B.Ar) × 100 × 365. All measurements were in accordance with a standard protocol [18].

#### 2.6.2. Bone Modeling

Periodontal ligament attached bone surfaces were selected to determine whether bone modeling was affected by OVX. The parameters including MAR and BFR (*μ*m^3^/*μ*m^2^/day) were calculated as described above.

### 2.7. Statistical Analysis

All data were presented as mean values ± SD and analyzed by independent* t*-tests and nonparametric tests using IBM SPSS statistics software (version 19.0, IBM Crop, Armonk, NY, USA). The significance level was set as *P* < 0.05.

## 3. Results

### 3.1. Clinical Examination

General well-being was defined as normal daily food and water intake, unremarkable behavioral pattern, and normal daily activity of rats during entire experimental period.

### 3.2. Micro-CT Examination

**Figure 2:**
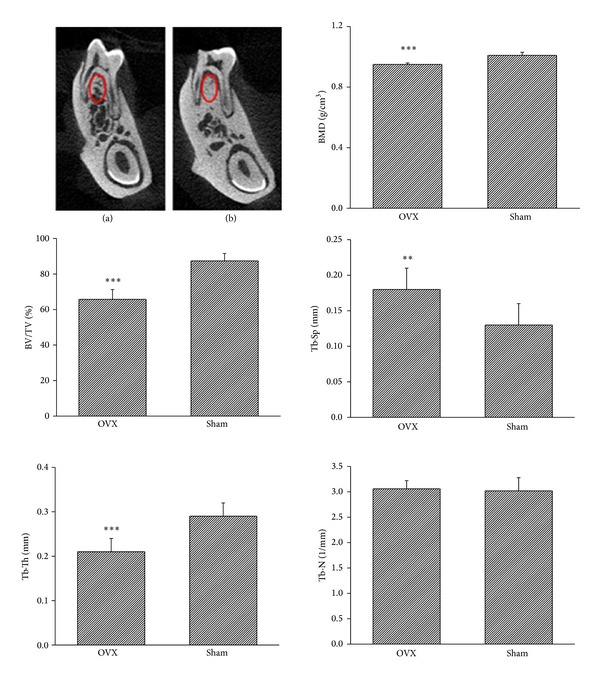
OVX changes the microarchitecture in rat mandibles. Micro-CT images show remarkable trabecular bone loss in OVX rats (a) compared with that in the control (b) group. BMD and BV/TV in OVX group are significantly decreased compared to those in control group. Tb*·*Th decreases and Tb*·*Sp increases significantly in OVX group. Tb*·*N shows no significant difference in both groups. BMD: bone mineral density; BV/TV: bone volume/tissue volume; Tb.Th: trabecular thickness; Tb.N: trabecular number; and Tb.Sp: trabecular separation. **P < 0.01 and ***P < 0.001.

In the control group, the trabecular bone presented as a well-connected network, while in the OVX group the trabecular bone withered and became separated. Quantitative analysis revealed that the BMD (*P* < 0.001) and percentage of bone volume (BV/TV, *P* < 0.001) were significantly decreased in OVX rats compared to those of the control group. The trabecular thickness decreased (*P* < 0.001) whereas the trabecular separation increased (*P* < 0.01) significantly in rats of OVX group. There was an increase in the number of trabeculae in OVX rats but the difference was not significant (*P* = 0.75) (Figure 2).

### 3.3. Histomorphometric Assessment

#### 3.3.1. Intracortical Remodeling

**Figure 3:**
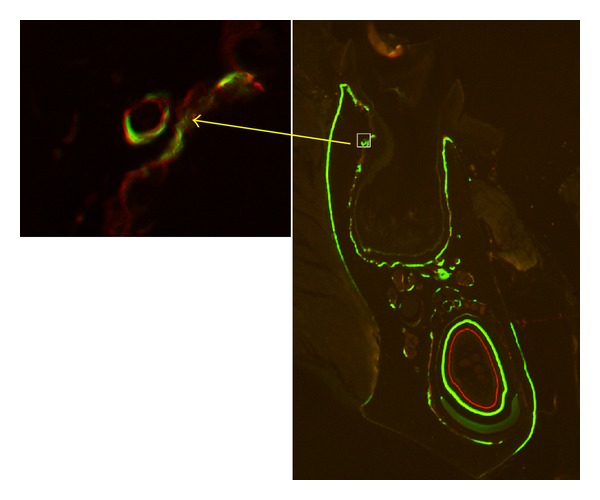
Photomicrograph of mandible in OVX rats viewed with fluorescent light. Labeled osteons (indicated by arrow) are found.

**Figure 4:**
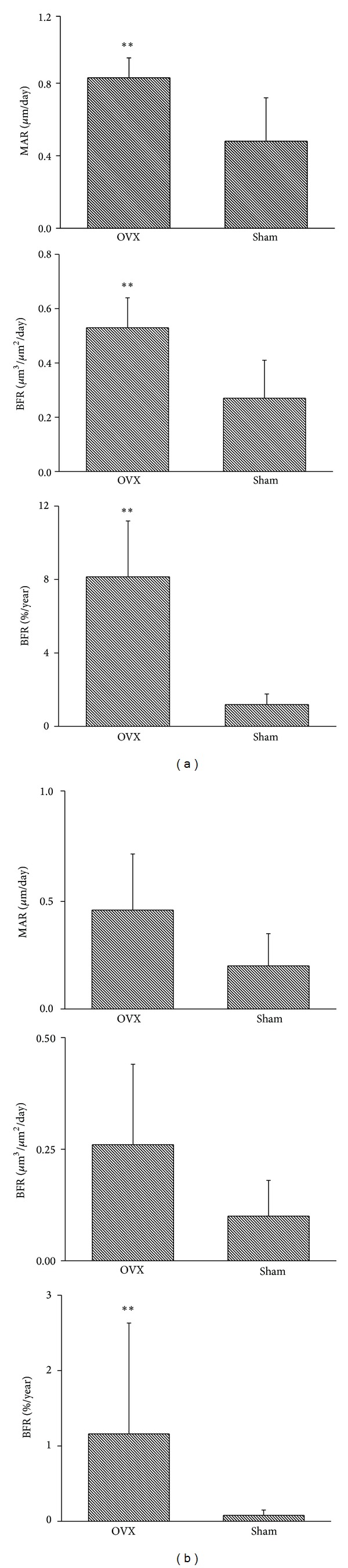
Ovariectomy stimulates remodeling activity in mandible of rat. In the alveolar cortical region (a), MAR, surface-based BFR (*μ*m^3^/*μ*m^2^/day), and intracortical BFR (% year) are significantly higher in OVX rats than in the control group. In the nonalveolar region (b), intracortical BFR (% year) is significantly increased in OVX group, whereas MAR and surface-based BFR (*μ*m^3^/*μ*m^2^/day) show no significant differences. MAR: mineral apposition rate and BFR: bone formation rate. **P < 0.01.

In the alveolar bone region, there was no significant difference related to the bone area between the OVX rats (3.19 ± 0.73 mm^2^) and the control group (2.81 ± 0.48 mm^2^). Labeled osteons were found in all OVX rats (Figure 3), indicating activated bone remodeling in the area. The average number of labeled osteons in OVX rats (2.48 ± 1.03 per mm^2^) was significantly higher (*P* < 0.01) than the one in the control group (0.89 ± 0.43 per mm^2^). Histomorphometric assessment disclosed that intracortical MAR (*P* < 0.01), surface-based BFR (*P* < 0.01), and intracortical BFR (*P* < 0.01) were all significantly stimulated in OVX rats (Figure 4(a)).

In nonalveolar regions, no significant difference in the bone area between OVX rats (4.28 ± 0.38 mm^2^) and the control group (4.33 ± 0.22 mm^2^) was detected. The number of labeled osteons in OVX rats (0.43 ± 0.18 per mm^2^) was significantly higher (*P* < 0.05) than in the control group (0.19 ± 0.17 per mm^2^). Compared to the alveolar bone region, the number of labeled osteons was much less than in the nonalveolar bone region in both OVX rats (2.48 ± 1.03 versus 0.43 ± 0.18 per mm^2^) and the control group (0.89 ± 0.43 versus 0.19 ± 0.17 per mm^2^). In OVX rats, intracortical BFR was significantly increased compared to the control group. Regarding MAR and surface-based BFR, no significant difference between OVX rats and the control group was found (Figure 4(b)).

#### 3.3.2. Bone Modeling

The MAR (1.47 ± 0.47 *μ*m/day) and surface-based BFR (0.99 ± 0.36 *μ*m^3^/*μ*m^2^/day) in OVX rats were significantly higher (*P* < 0.01) in the surface adjacent to the periodontal ligament compared to those in the control group (0.53 ± 0.11 *μ*m/day; 0.16 ± 0.05 *μ*m^3^/*μ*m^2^/day).

## 4. Discussion

Primates and dogs represent ideal animal models to investigate diseases characterized by abnormal intracortical remodeling. Both models show intracortical remodeling throughout the skeleton, just like humans [13, 19]. Ethical issues together with high expenses, however, do restrict their laboratory use. Rodents have been used instead to study these conditions, but their model value is limited because these animals usually do not display intracortical remodeling [13].

Some studies reported that intracortical remodeling in rodents could be stimulated under pathological conditions [8–10]. These findings raised the interest of using a “modified” rodent model mimicking human bone conditions to investigate various bone diseases. Some studies have demonstrated that ovariectomy remarkably stimulated intracortical remodeling in long bones of rats and mice [8]; however, the influence of ovariectomy on jawbones has not yet been fully understood.

Jawbones have a unique structure and undergo the highest intracortical remodeling rate throughout the skeleton [11, 20]. Many diseases associated with abnormal intracortical remodeling, such as BRONJ, affect the alveolar bone only [21]. Therefore it is of great interest to establish a rodent model which mimics the intracortical bone remodeling of the human skeleton.

This study investigated radiographic and histomorphometric changes in mandibular cancellous and cortical bones of OVX rats. Consistent with previous studies, both trabecular bone volume and bone mineral density decreased significantly after surgery [22, 23].

Histomorphometry is considered to be the gold standard in dynamic bone research. Better than other methods which estimate the remodeling status detecting humeral biomarkers, it may provide site-specific information directly by measuring MAR and BFR at different time points using the fluorochrome sequential labeling approach [15].

As mature mammals routinely undergo remodeling in cortical bones, the place where bone resorption and bone formation occur simultaneously, BFR evaluation might provide insight into the intracortical remodeling rate. In this study, active intracortical bone remodeling characterized by labeled osteons in the cortical bone region was found in both OVX rats and the control group. Rats after sham surgery manifested a very low-rate mandibular remodeling, whereas OVX rats disclosed significantly increased remodeling rates. In the alveolar region, ovariectomy stimulated a six-time higher intracortical BFR compared with that in control animals. These findings were similar to a previous report by Kubek et al. which so far is the only study addressing this issue in mandibles in a mouse model [10].

In the bone surface attached to the periodontal ligament, where bone modeling primarily takes place [24], a significantly increased bone formation rate was detected in OVX rats. On the contrary to this study, Kubek et al. [10] did not find any significant increase in bone formation. This discrepancy may be attributed to the difference of animal species (C3H mice versus SD rat) and/or the length of the experiment, 8 weeks versus 18 weeks after surgery. Early after ovariectomy, the bone resorption surpasses the bone formation leading to bone loss; thereafter both processes reach a steady state. The time needed to reach this steady state varies in rats between 90 and 270 days [25]. To ensure that the analysis was performed during the steady state, a long experimental period of 18 weeks was selected in the here presented study.

The study of Kubek et al. [10] confirmed that OVX mice represented a useful tool to study BRONJ. Compared to mice, rats might represent a more versatile animal model in the research field of BRONJ as they also offer other investigations related to bone remodeling processes such as ligature induced-periodontal diseases or pulp-exposure induced periapical infections inducing BRONJ. Such conditions are technically difficult to be mimicked in mice because of the smaller size of teeth and jawbones.

## 5. Conclusions

This study demonstrated that ovariectomy can stimulate intracortical remodeling in rat jawbones, to the authors' knowledge, for the first time. It might be taken into consideration that OVX rats are useful to study various bone diseases associated with abnormal intracortical bone remodeling processes, such as BRONJ.
